# Supplementary material for: Gradient design of imprinted anode for stable Zn-ion batteries
Source: Nat Commun. 2023 Feb 6;14:641. doi: 10.1038/s41467-023-36386-3 (PMC9902526; doi:10.1038/s41467-023-36386-3)
Supplement: Supplementary file 1 — Supplementary Information [file 41467_2023_36386_MOESM1_ESM.pdf]

Supplementary Information

Gradient Design of Imprinted Anode for Stable Zn-ion Batteries

Qinghe Cao[1,2,3,4], Yong Gao[1,2,4], Jie Pu[1,2,4], Xin Zhao[1], Yuxuan Wang[1], Jipeng Chen[1], Cao Guan[1,2*]

[1]Institute of Flexible Electronics, Northwestern Polytechnical University, Xi'an 710072, China

[2]Key laboratory of Flexible Electronics of Zhejiang Province, Ningbo Institute of Northwestern Polytechnical University, 218 Qingyi Road, Ningbo, 315103, China.

[3]Department of Materials Science and Engineering, National University of Singapore, Singapore 117576, Singapore

[4]These authors contribute equally: Qinghe Cao, Yong Gao, Jie Pu.

Corresponding Authors

*E-mail: iamcguan@nwpu.edu.cn (C. Guan)

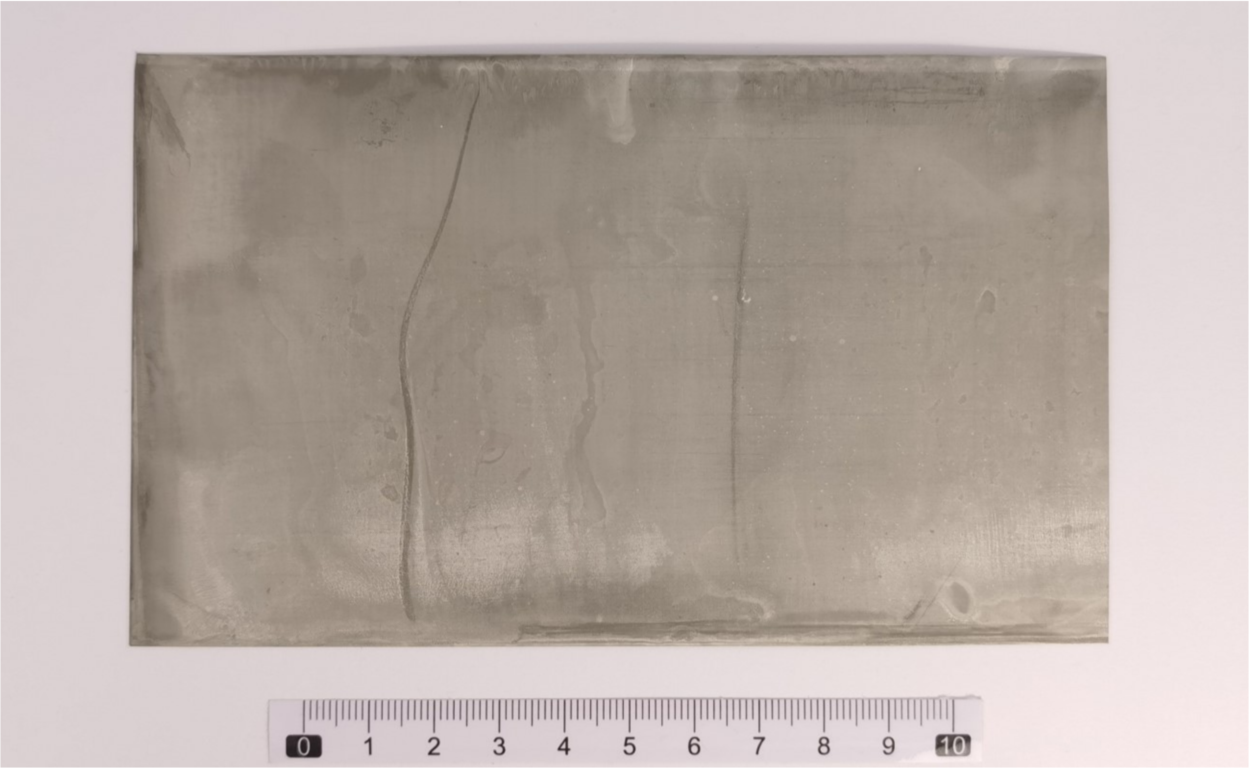

Supplementary Figure 1. Optical photo of PVDF-Sn@Zn gradient electrode with a large size of 10 × 15 cm^2.

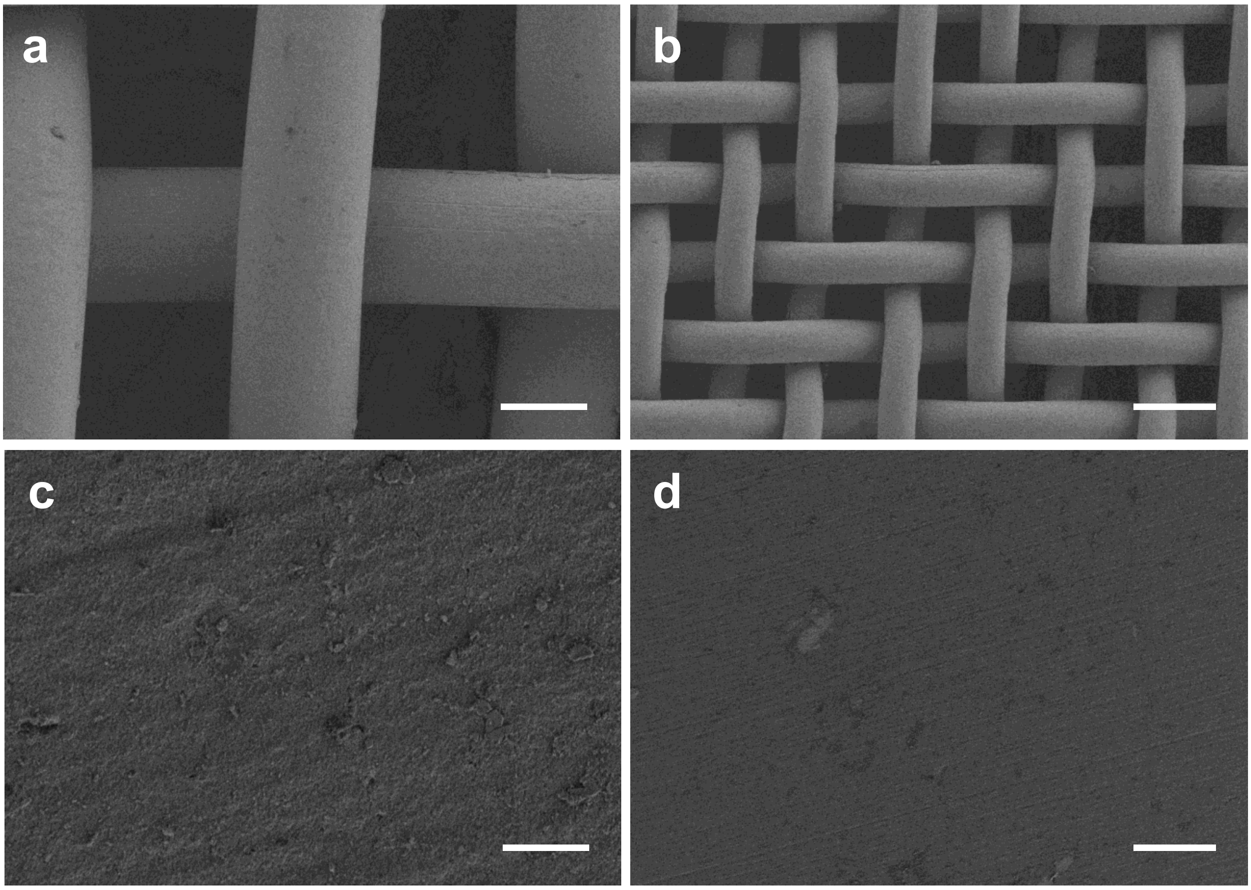

Supplementary Figure 2. SEM images of **a, b** SSM and **c, d** Sn@Zn. Scale bar, 15 μm for **a, c** and 60 μm for **b, d**.

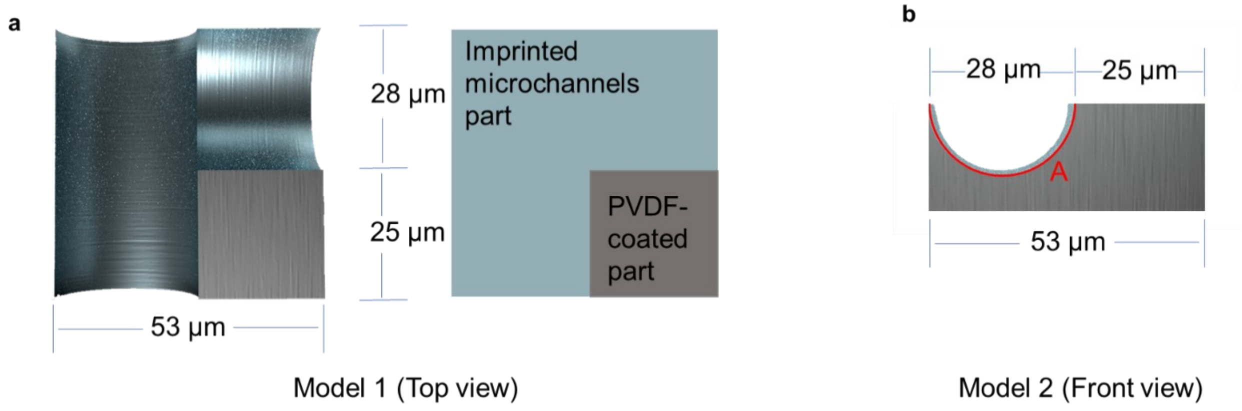

Supplementary Figure 3. a Top (Model 1) and **b** front (Model 2) view of the imprinted electrode.

Assuming that the PVDF-coated areas are inactive and the imprinted microchannels are active, Model 1 (Supplementary Figure 3a, top view) is developed to calculate the enhanced area of PVDF-Sn@Zn gradient electrode than planar electrode. According to the stainless steel mesh (SSM) parameters and SEM images (stainless steel wire diameter of 28 μm and pore diameter of 25 μm), the percentage of PVDF coated part on the whole electrode are about 22.2% ($25^2/(25+28)^2*100\%$). Thus, the imprinted microchannels account for 77.8% (1-22.2%).

To obtain the increased area of the microchannel structure compared to the planar one, Model 2 (Supplementary Figure 3b, front view) is built, where the perimeter of cross-section A is π*28 (assuming that half of each SSM wire is imprinted into the Zn metal). Therefore, the area of half a cylinder is about π*28/2*l, by fixing length as a unit one. The area of a planar electrode under the same conditions is about 28*1. Thus, it can be obtained that the active area of the PVDF-Sn@Zn electrode in the microchannels increases by about 57.1% (π/2-1). Accordingly, the ratio of the active area of the PVDF-Sn@Zn electrode can be calculated as 122.2% (77.8%*1.571), which means that the active area of the PVDF-Sn@Zn electrode is roughly 22.2% higher than that of the planar electrode.

In addition, for electric field, it is ideal to assume that the entire electric field is concentrated in the microchannels. According to the electric field simulation, there is still a part of the electric field in the PVDF-coated areas, which can share the electric field intensity. The overall area of the electrode after imprinting is increased by 44.4% (77.8%*57.1%) compared with the planar electrode. Therefore, it is beneficial to reduce the electric field intensity of the whole electrode.

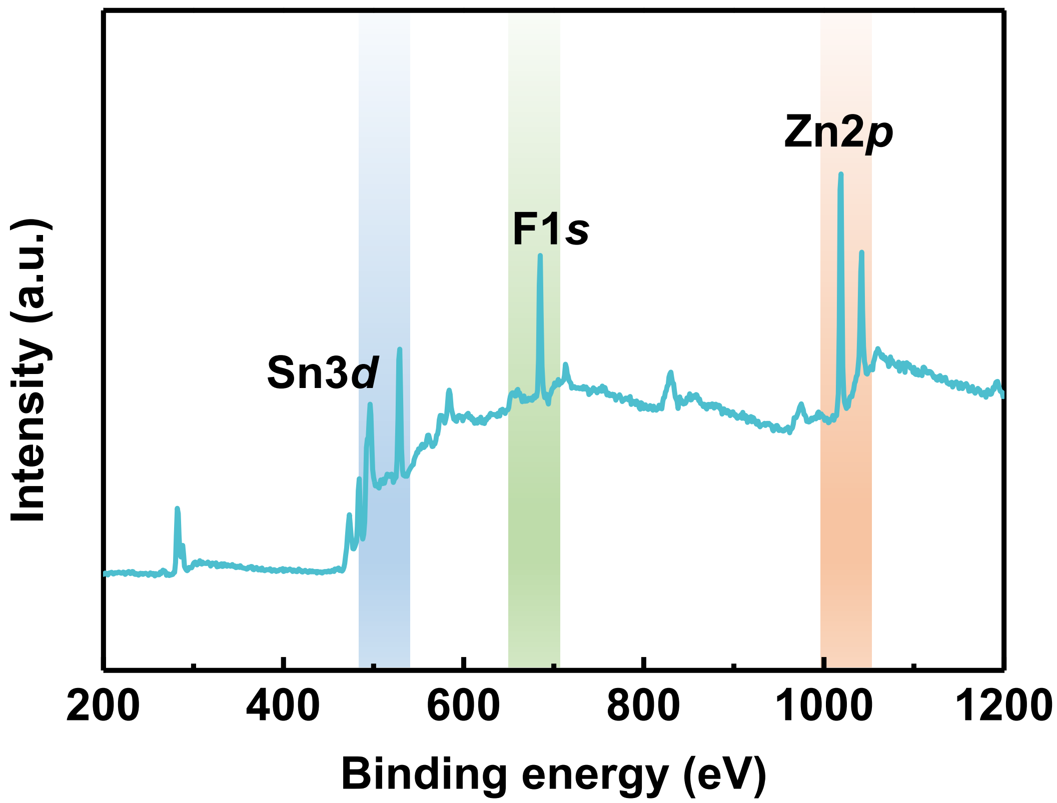

Supplementary Figure 4. XPS spectra of PVDF-Sn@Zn gradient electrode.

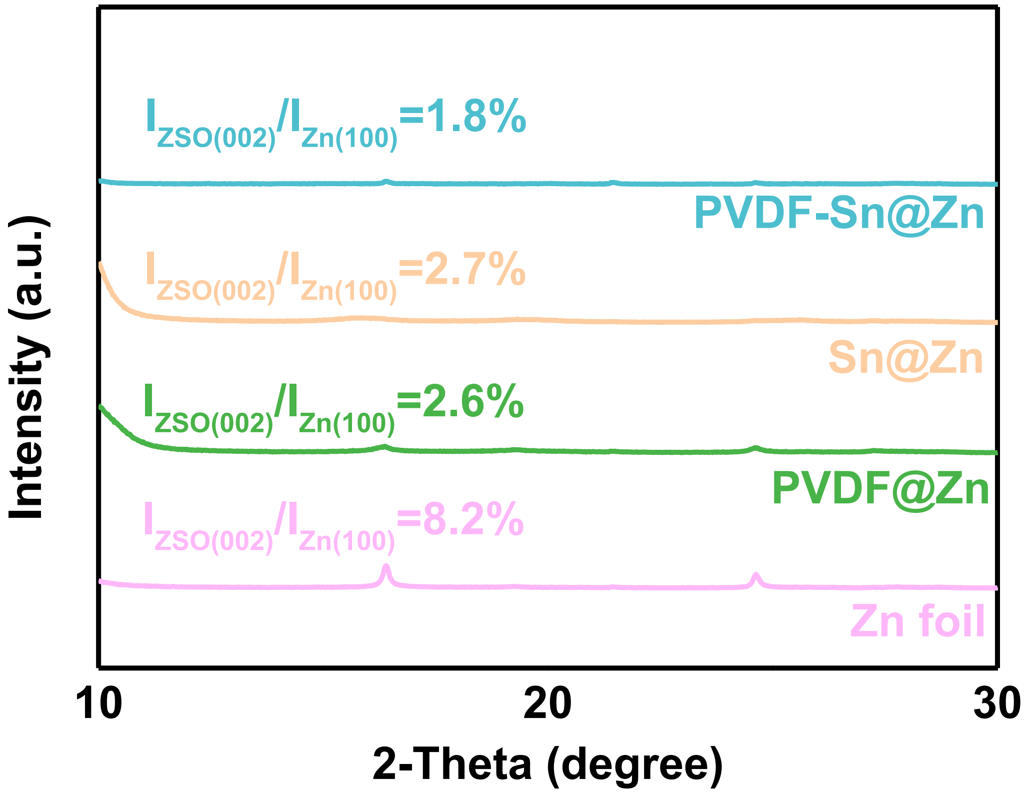

Supplementary Figure 5. XRD patterns of different electrodes after immersion in 2 M $ZnSO_4$ electrolyte for 7 days.

As shown in Supplementary Figure 5, the PVDF-Sn@Zn gradient electrode shows a weak diffraction peak of $Zn_4SO_4(OH)_6 \cdot xH_2O$ (ZSO), which proves to be effective in suppressing the formation of by-products. To better compare the corrosion resistance of the different electrodes, we compared the ratio of intensity of by-products to Zn metal ($I_{ZSO(002)}/I_{Zn(100)}$), where the values of Zn foil, PVDF@Zn, Sn@Zn and PVDF-Sn@Zn gradient electrodes are 8.2%, 2.6%, 2.7% and 1.8%, respectively. The results further demonstrate the good corrosion resistance of the PVDF-Sn@Zn gradient electrode.

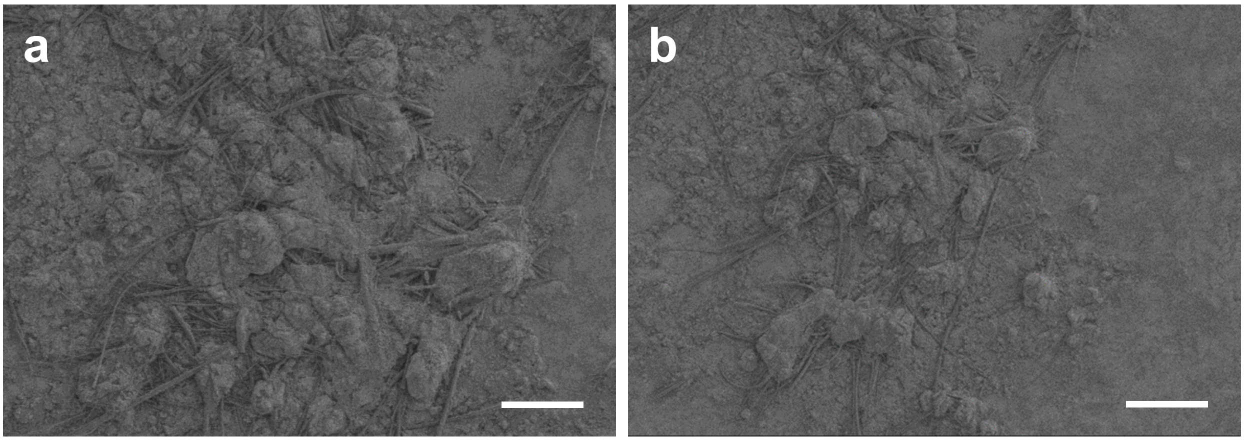

Supplementary Figure 6. SEM images of Zn foil after Zn deposition with a capacity of 5 mAh cm^{-2}.

Scale bar, 30 μm for **a** and 60 μm for **b**.

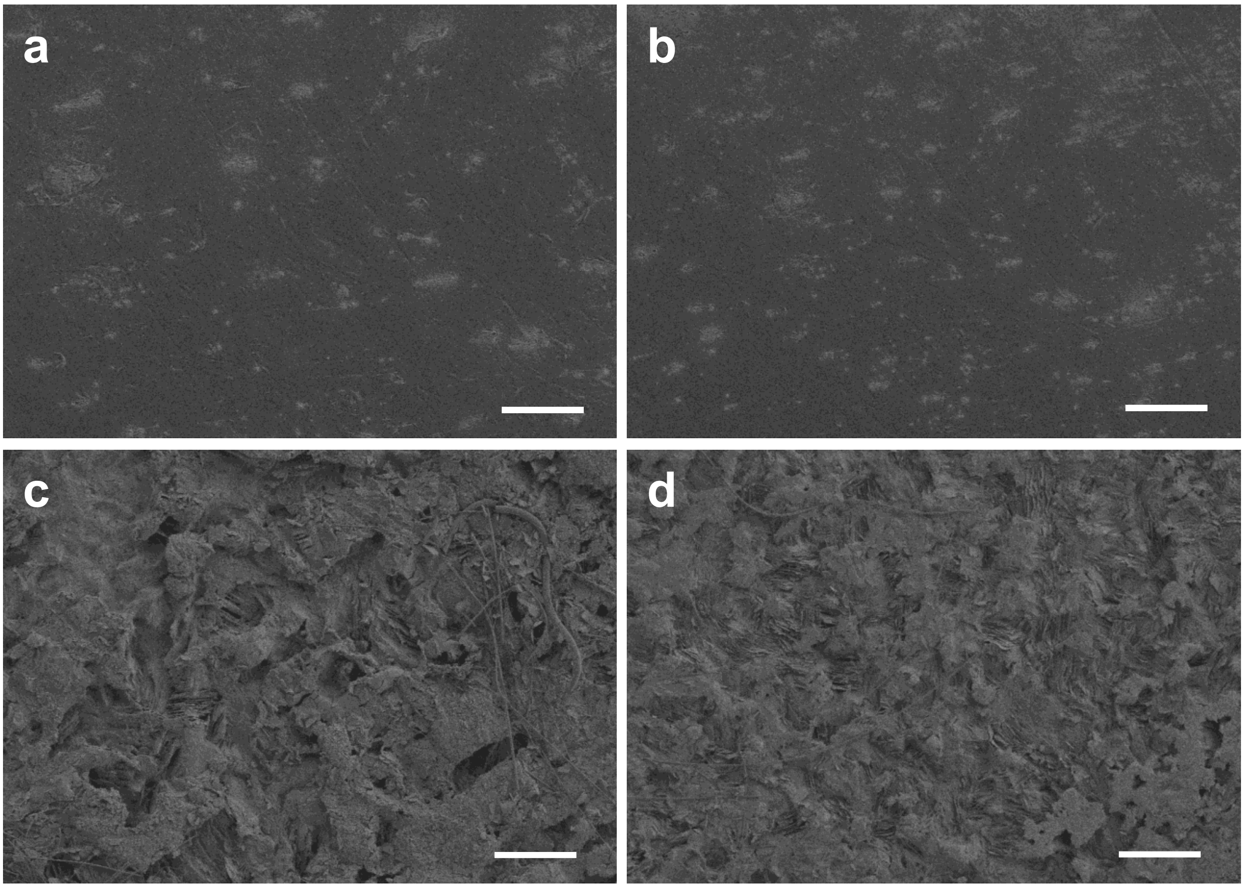

Supplementary Figure 7. SEM images of PVDF@Zn after Zn deposition with capacities of **a, b** 5 mAh cm^{-2} and **c, d** 10 mAh cm^{-2}. Scale bar, 30 μm for **a, c** and 60 μm for **b, d**.

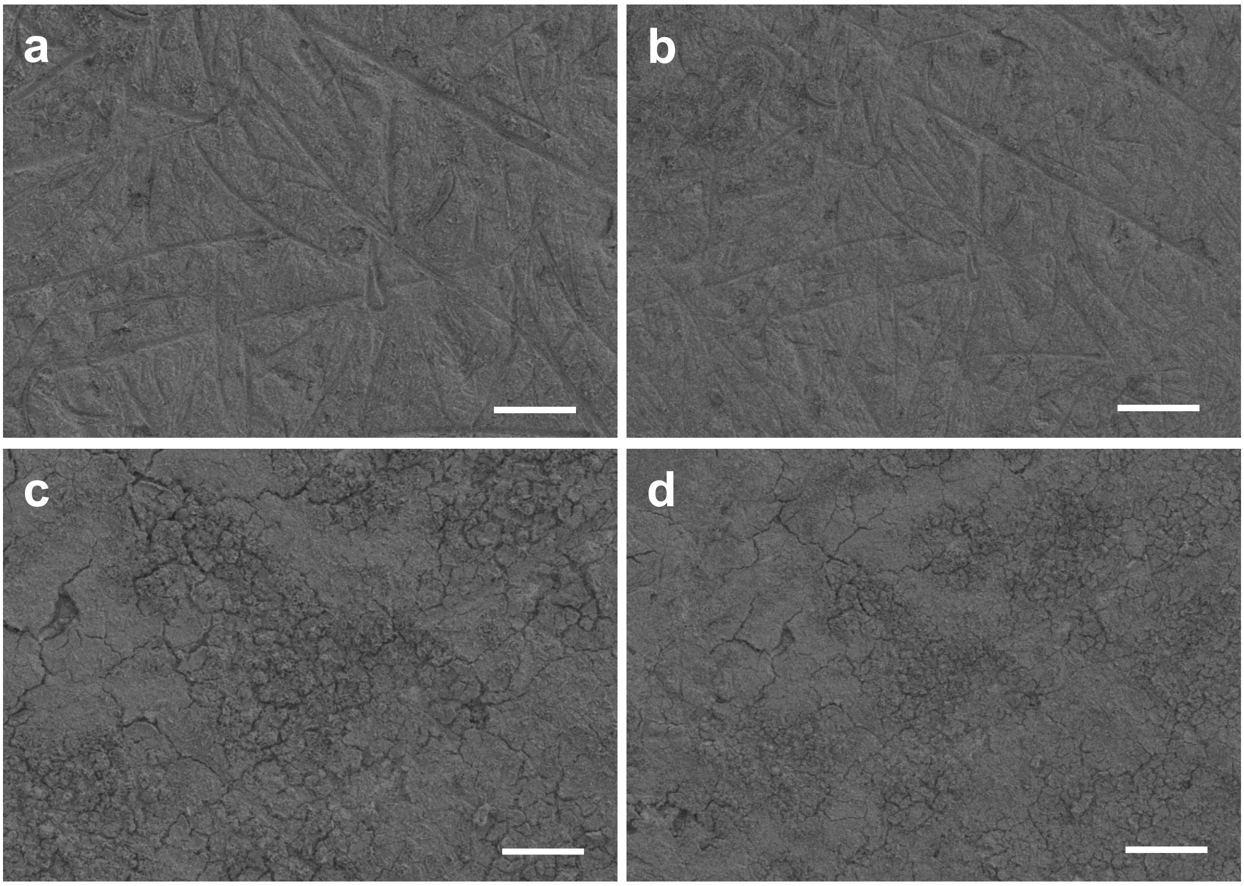

Supplementary Figure 8. SEM images of Sn@Zn after Zn deposition with capacities of **a, b** 5 mAh cm^{-2} and **c, d** 10 mAh cm^{-2}. Scale bar, 30 μm for **a, c** and 60 μm for **b, d**.

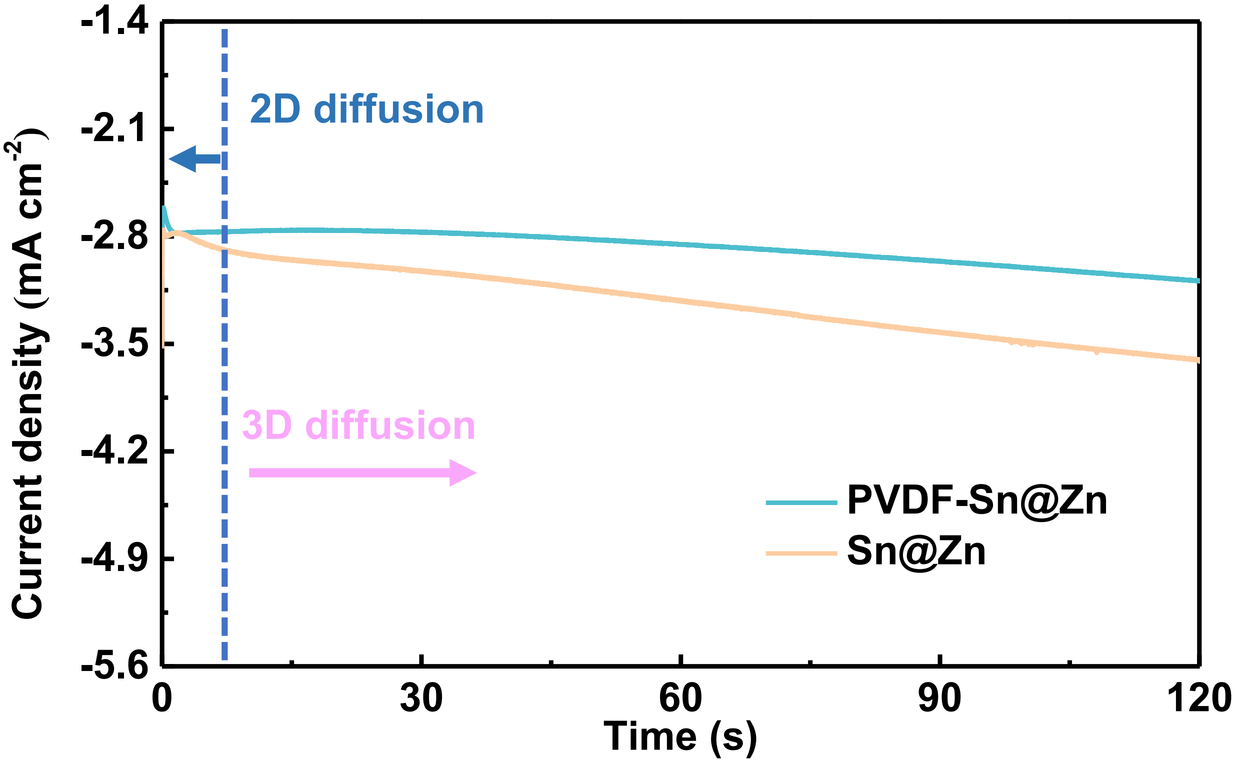

Supplementary Figure 9. Chronoamperometry (CA) curves of Sn@Zn and PVDF-Sn@Zn gradient electrodes at a constant potential of -30 mV.

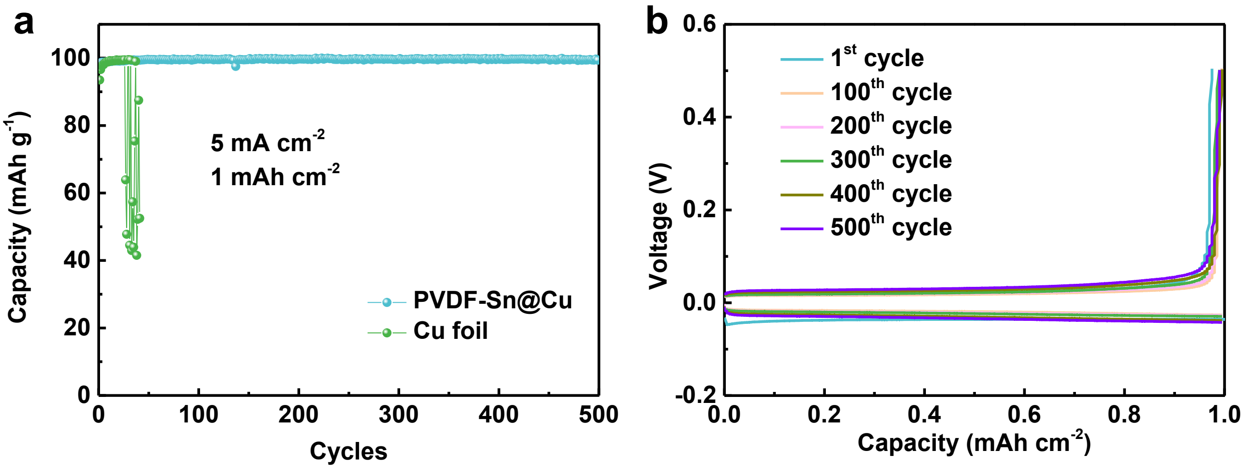

Supplementary Figure 10. a Coulombic efficiency of PVDF-Sn@Cu//Zn and Cu foil//Zn cell at current density of 5 mA cm^{-2} and capacity of 1 mAh cm^{-2}. **b** Voltage profiles of PVDF-Sn@Cu//Zn cell at the 1st, 100th, 200th, 300th, 400th and 500th cycles.

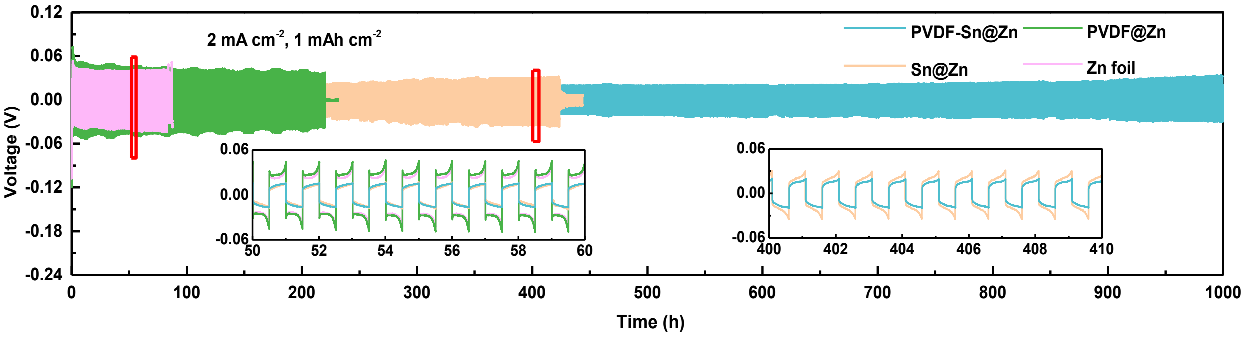

Supplementary Figure 11. Voltage profiles of Zn//Zn symmetric cells at current density/capacity of 2 mA cm^{-2}/1 mAh cm^{-2}.

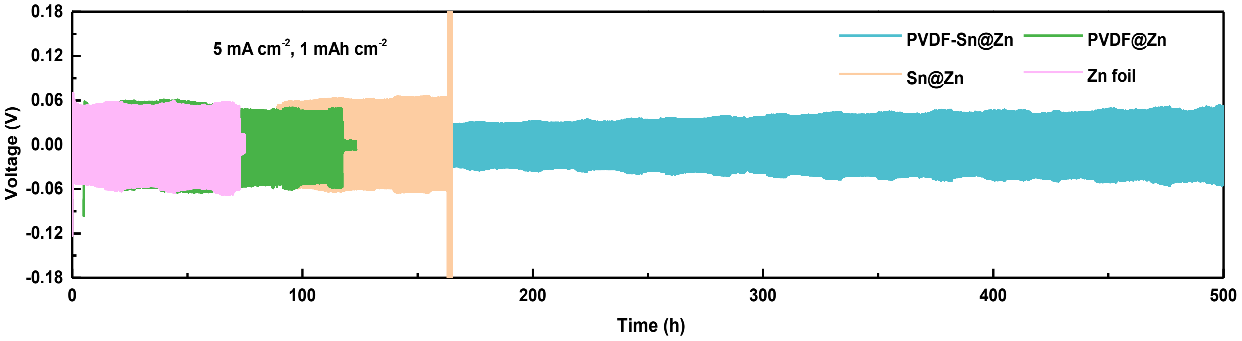

Supplementary Figure 12. Voltage profiles of Zn//Zn symmetric cells at current density/capacity of 5 mA cm^{-2}/1 mAh cm^{-2}.

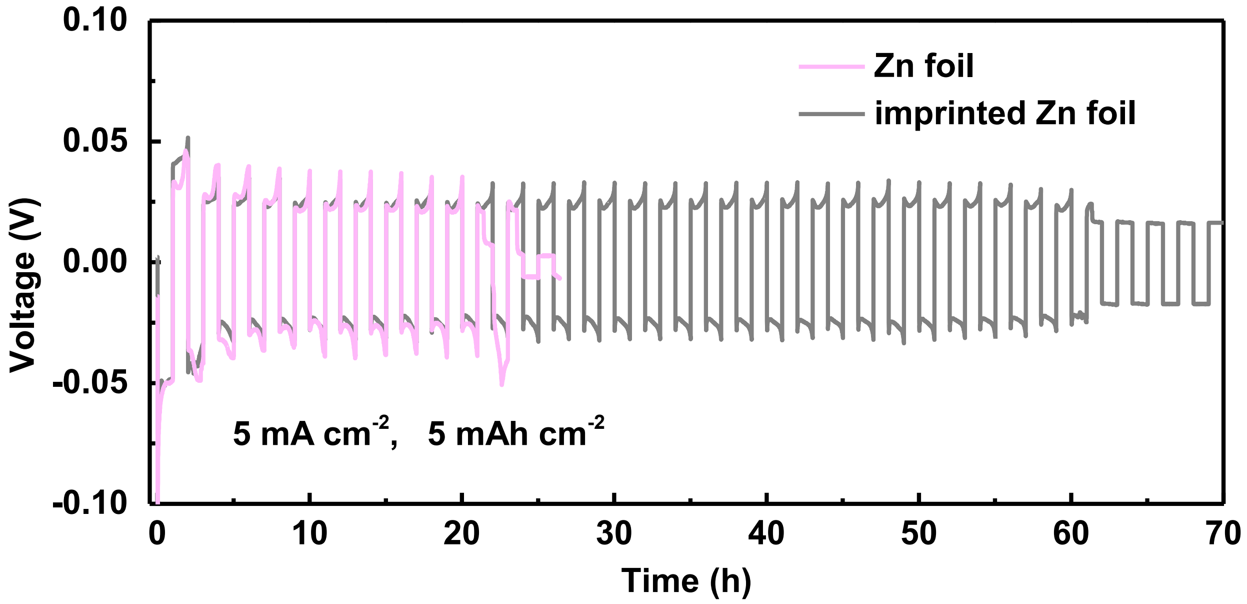

Supplementary Figure 13. Voltage profiles of Zn//Zn symmetric cells at current density/capacity of 5 mA cm^{-2}/5 mAh cm^{-2}.

The cycling performance of Zn//Zn symmetric cells based on imprinted Zn foil (noted as imprinted Zn foil//imprinted Zn foil) is measured. The imprinted Zn foil//imprinted Zn foil cell shows smaller voltage hysteresis and better cycling performance than the Zn foil//Zn foil cell, benefiting from the increased surface area and void spaces from the imprinting process. However, the imprinted Zn foil//imprinted Zn foil cell still shows a rapid voltage decay at about 61 h, which can be attributed to a short circuit caused by the growth of dendrites at the top of the electrode.

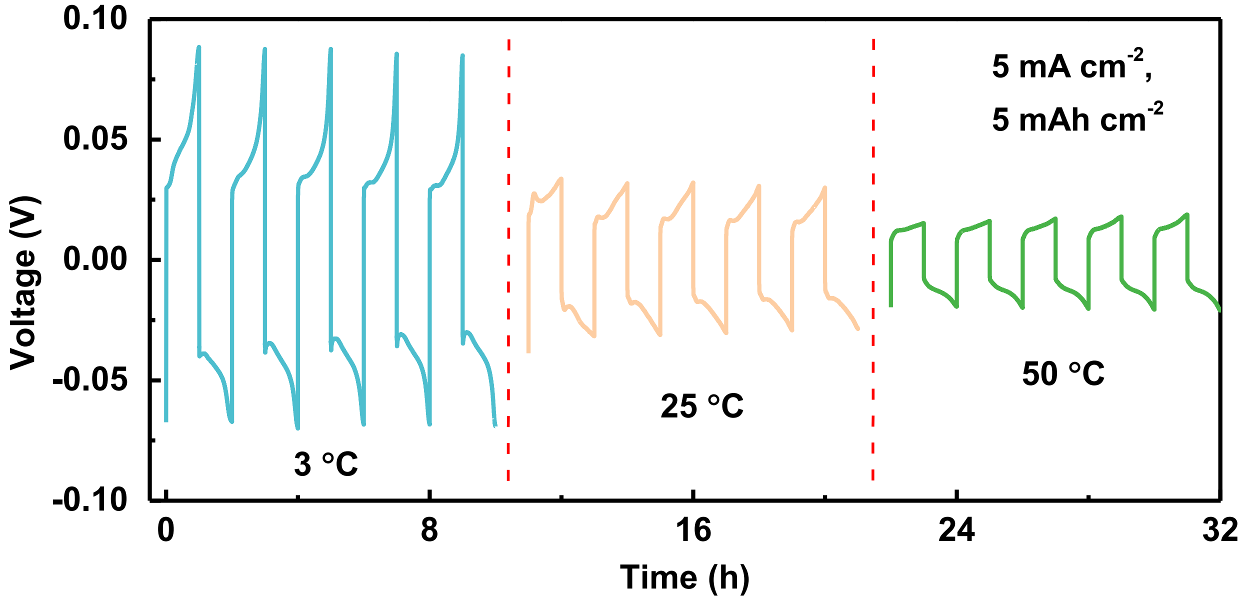

Supplementary Figure 14. Voltage curve for PVDF-Sn@Zn//PVDF-Sn@Zn symmetric cells with current density/capacity of 5 mA cm^{-2}/5 mAh cm^{-2} at different temperatures.

Changes in the temperature of the test environment can have a significant effect on voltage hysteresis. To explore the effect of temperature on voltage hysteresis, the voltage-time curves of PVDF-Sn@Zn//PVDF-Sn@Zn cell at different temperatures were investigated. As shown in Supplementary Figure 14, PVDF-Sn@Zn//PVDF-Sn@Zn cell exhibits voltage hysteresis of approximately 85.5 mV, 29.5 mV and 17.0 mV at 3°C, 25°C and 50°C, respectively, demonstrating that increased ambient temperature may be a significant contributor to reduced voltage hysteresis.

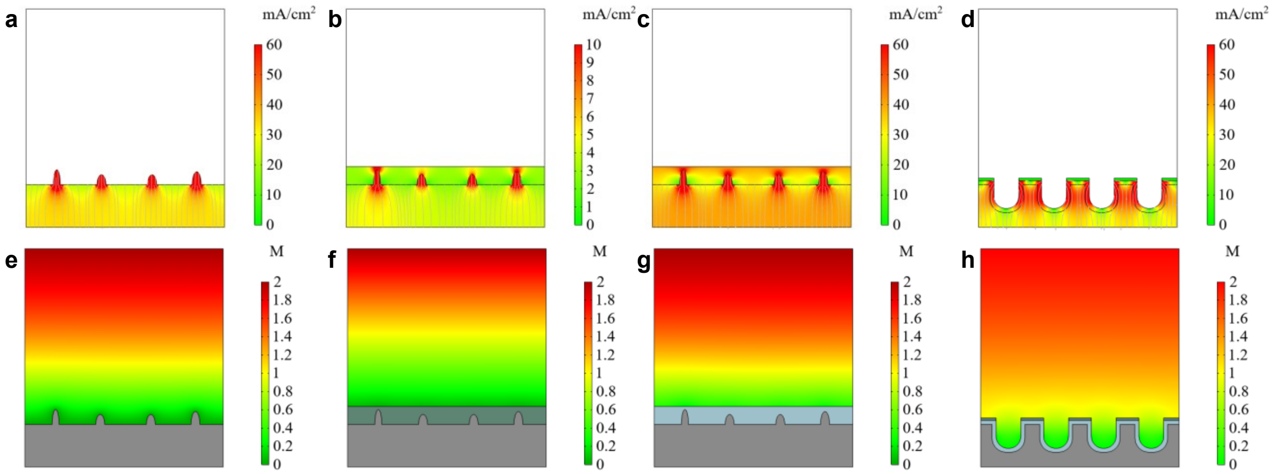

Supplementary Figure 15. Current density simulation on **a** Zn foil, **b** PVDF@Zn, **c** Sn@Zn and **d** PVDF-Sn@Zn gradient electrodes. Zn^{2+} ion concentration distribution simulation on **e** Zn foil, **f** PVDF@Zn, **g** Sn@Zn and **h** PVDF-Sn@Zn gradient electrodes.

To reveal the advantages of the PVDF-Sn@Zn gradient anode over than the other three controlled electrodes, simulations of the current density and Zn^{2+} ion concentration distribution are studied.

Current density distribution indicates that the roughness of Zn foil would result in the concentrated current density on the bumps, which causes severe polarization (Supplementary Figure 15a). The non-conductivity of the PVDF layer can effectively reduce the current density on the electrode surface, but it is still uneven in these bumps (Supplementary Figure 15b). The Sn layer with good electrical conductivity also cannot solve the problem, with current density accumulated in bumps and resulting in uneven Zn deposition (Supplementary Figure 15c). In comparison, the non-conductive PVDF layer on the top of the PVDF-Sn@Zn gradient electrode can effectively reduce the current density at the top surface of the electrode, thereby inducing the preferential deposition of Zn in the imprinted microchannels and improving the cycle performance (Supplementary Figure 15d).

The Zn^{2+} ion concentration distribution shows that the Zn^{2+} ion concentration on the Zn foil is low due to its unsatisfactory Zn affinity and high nucleation overpotential (Supplementary Figure 15e). The non-conductive and hydrophobic PVDF will further reduce the Zn^{2+} ion concentration on the surface of Zn metal (Supplementary Figure 15f). The Sn layer can increase the Zn^{2+} ion concentration of the Zn/electrolyte interface due to its good zincophilicity and hydrophilicity, thus can effectively improve the Zn deposition uniformity (Supplementary Figure 15g). PVDF-Sn@Zn gradient electrode benefits from the advantages of the 3D structure, which can accelerate the diffusion of Zn^{2+} ions and increase the Zn^{2+} ion concentration on the electrode surface. In addition, the gradient design induces the Zn^{2+} ion concentration incline to microchannels, resulting in uniform deposition of Zn in the microchannels (Supplementary Figure 15h).

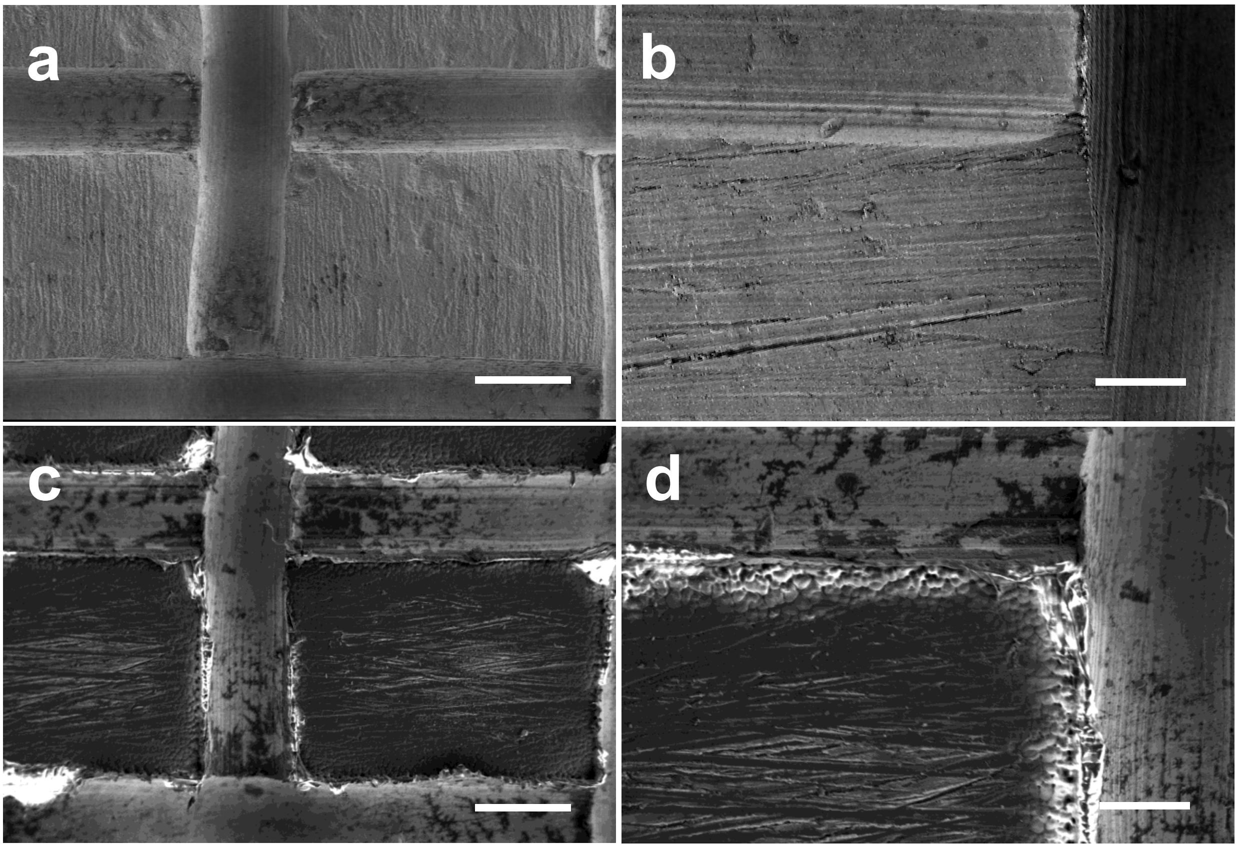

Supplementary Figure 16. SEM images of **a,b** imprinted Sn@Zn and **c,d** PVDF-Sn@Zn gradient electrodes (prepared with 80-mesh SSM). Scale bar, 100 μm for **a,c** and 50 μm for **b,d**.

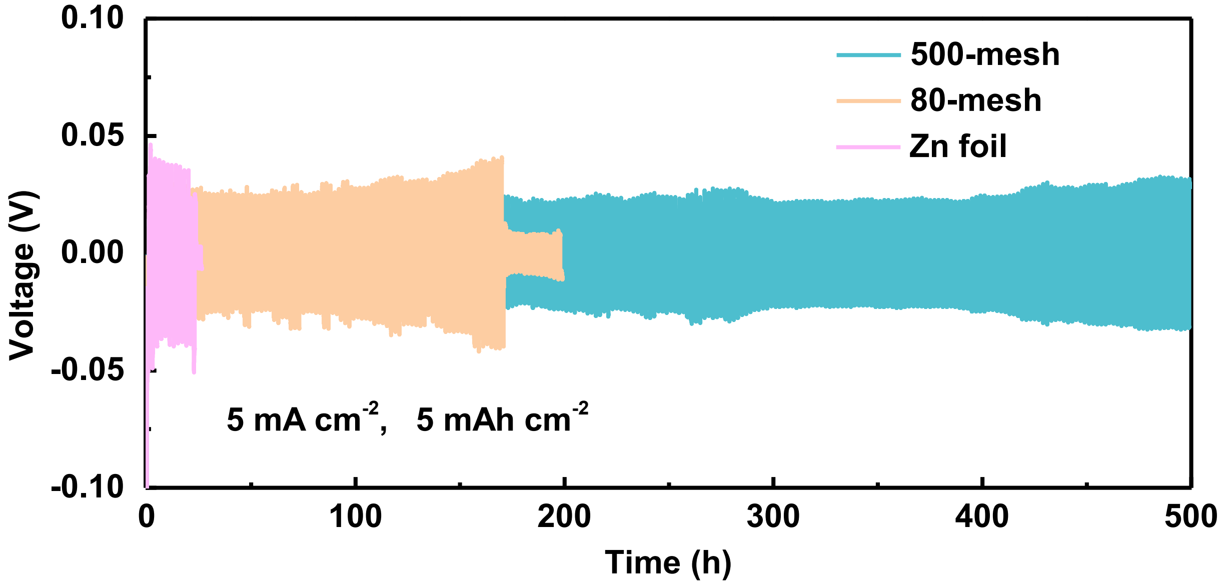

Supplementary Figure 17. Voltage profiles of Zn//Zn symmetric cells at current densities/capacities of 5 mA cm^{-2}/5 mAh cm^{-2}.

The cycling performance shows a sudden drop in voltage at about 171 h for PVDF-Sn@Zn 80-mesh//PVDF-Sn@Zn 80-mesh, indicating a short circuit in the cell. The relatively poor cycling performance of the electrode can be attributed to the smaller imprinted area and void space ratio, which limits the deposition modulation property and storage capacity of the electrode. We have also attempted to prepare PVDF-Sn@Zn gradient electrodes with 800-mesh SSM. However, during the rolling process, the 800-mesh SSM is easily peeled off from the Zn foil and cannot be maintained for the following coating procedure, therefore it could not be used to prepare gradient electrodes.

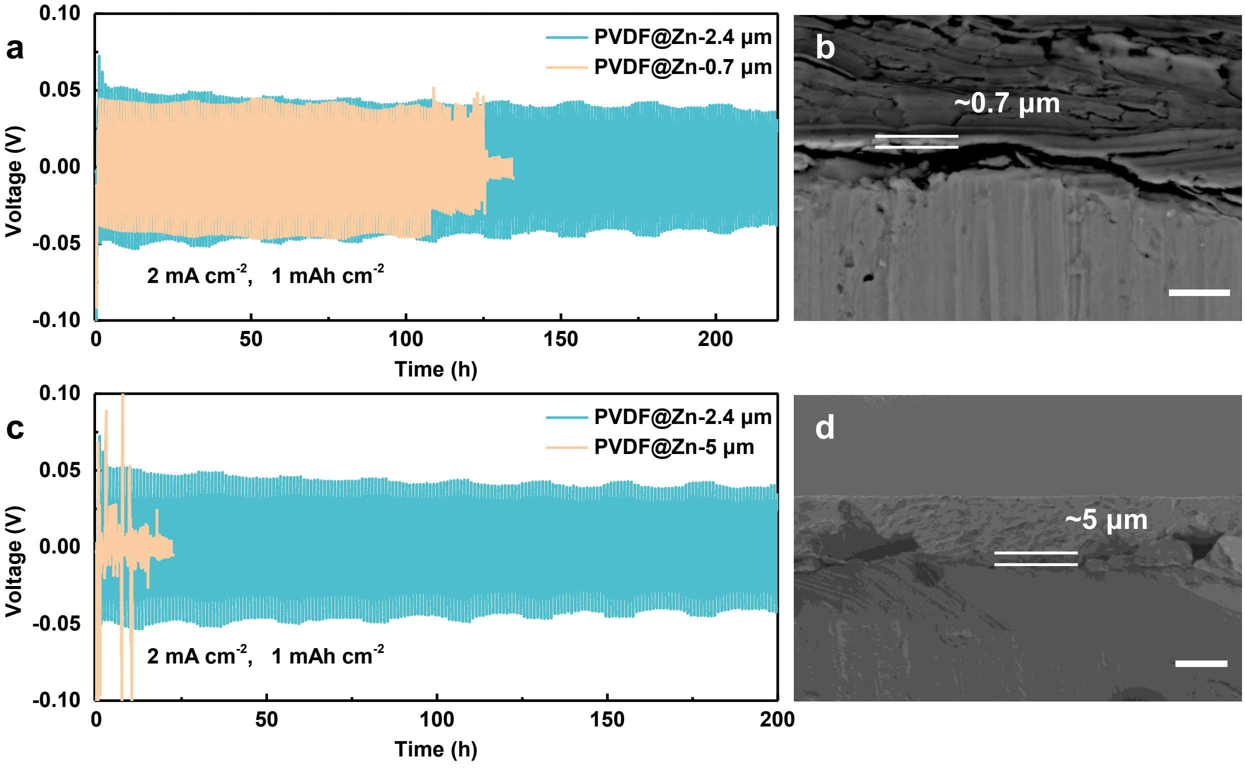

Supplementary Figure 18. a Voltage profiles of PVDF@Zn-0.7 μm//PVDF@Zn-0.7 μm symmetric cell at current density/capacity of 2 mA cm^{-2}/1 mAh cm^{-2}. **b** Cross-sectional SEM image of PVDF@Zn-0.7 μm. **c** Voltage profiles of PVDF@Zn-5 μm//PVDF@Zn-5 μm symmetric cell at current density/capacity of 2 mA cm^{-2}/1 mAh cm^{-2}. **b** Cross-sectional SEM image of PVDF@Zn-5 μm. Scale bar, 3 μm for **b** and 20 μm for **d**.

The PVDF thickness is optimized by comparing the cycling performance of different PVDF@Zn electrodes with different PVDF thicknesses. PVDF@Zn-0.7 μm electrode with a PVDF thickness of 0.7 μm is prepared as shown in Supplementary Figure 18a and b. The cycling performance shows that the PVDF@Zn-0.7 μm//PVDF@Zn-0.7 μm cell exhibits a short circuit after about 126 h, which can be attributed to limited improvement in corrosion resistance and incomplete coating (the drying process resulted in cracks in the thin layer). PVDF@Zn-5 μm electrode with a PVDF thickness of 5

μm is also prepared as shown in Supplementary Figure 18c and d. The cycling performance shows that the PVDF@Zn-5 μm//PVDF@Zn-5 μm cell exhibits a kinetic short circuit after only several cycles, much worse than the PVDF@Zn-2.4 μm//PVDF@Zn-2.4 μm cell, which can be attributed to the increased Zn^{2+} ion transfer resistance caused by the unsatisfactory ionic conductivity of PVDF. It can be inferred that PVDF can effectively improve the corrosion resistance of the electrode, but a too-thick PVDF layer is not conducive to uniform Zn deposition on the electrode surface.

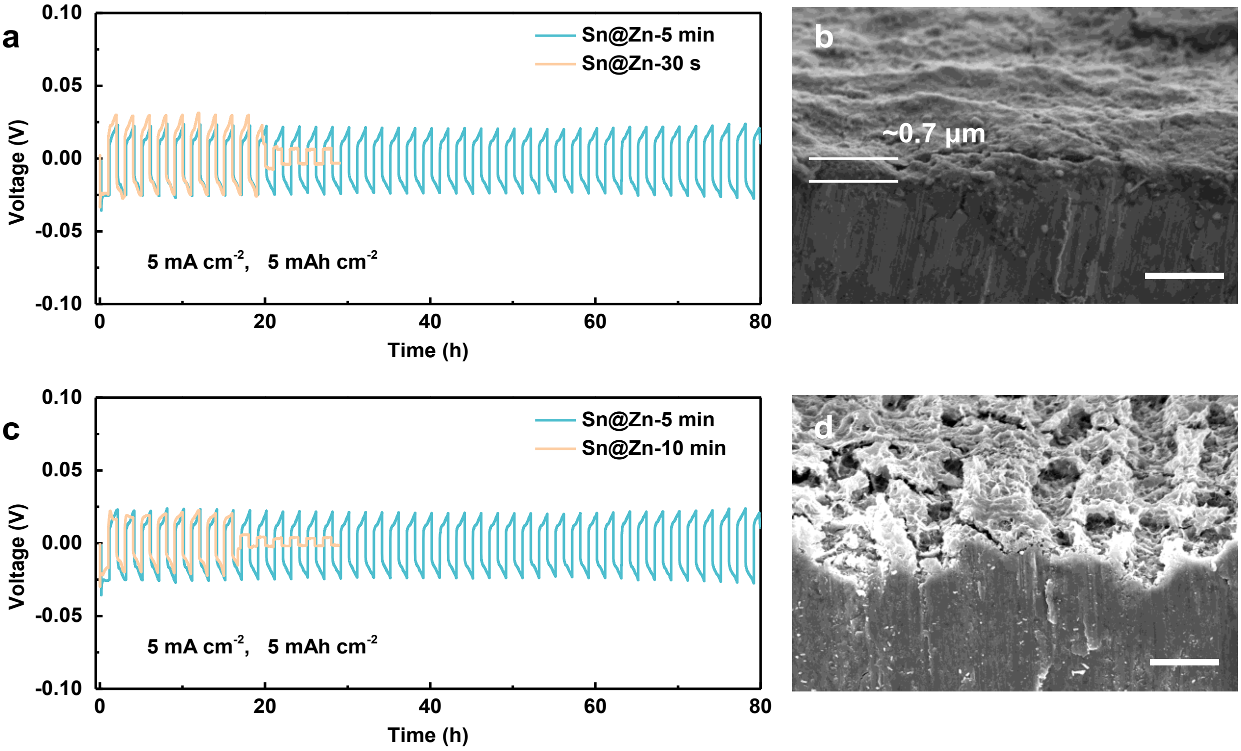

Supplementary Figure 19. a Voltage profiles of Sn@Zn-30 s//Sn@Zn-30 s symmetric cell at current density/capacity of 5 mA cm^{-2}/5 mAh cm^{-2}. **b** Cross-sectional SEM image of Sn@Zn-30 s. **c** Voltage profiles of Sn@Zn-10 min//Sn@Zn-10 min symmetric cell at current density/capacity of 5 mA cm^{-2}/5 mAh cm^{-2}. **d** Cross-sectional SEM image of Sn@Zn-10 min. Scale bar, 2 μm for **b** and 5 μm for **d**.

The optimization of the thickness of the Sn layer is carried out by controlling the immersion time of the Zn foil in the $SnCl_4$ solution. As shown in Supplementary Figure 19a and b. Sn@Zn-30 s electrode with Sn thickness of 0.7 um is prepared by placing Zn foil in $SnCl_4$ solution for 30 s. However, the cycling performance shows that the Sn@Zn-30 s//Sn@Zn-30 s cell experiences a short circuit after only about 20 h, which can be attributed to the limited modulation property of the thinner Sn layer. We also obtained a thicker Sn layer by soaking the Zn foil in $SnCl_4$ solution for 10 min, as shown in Supplementary Figure 19c and d. The Sn@Zn-10 min electrode shows a porous and non-uniform structure due to severe etching/reaction, and the Sn@Zn-10 min//Sn@Zn-10 min cell experiences a short circuit after only about 16 h.

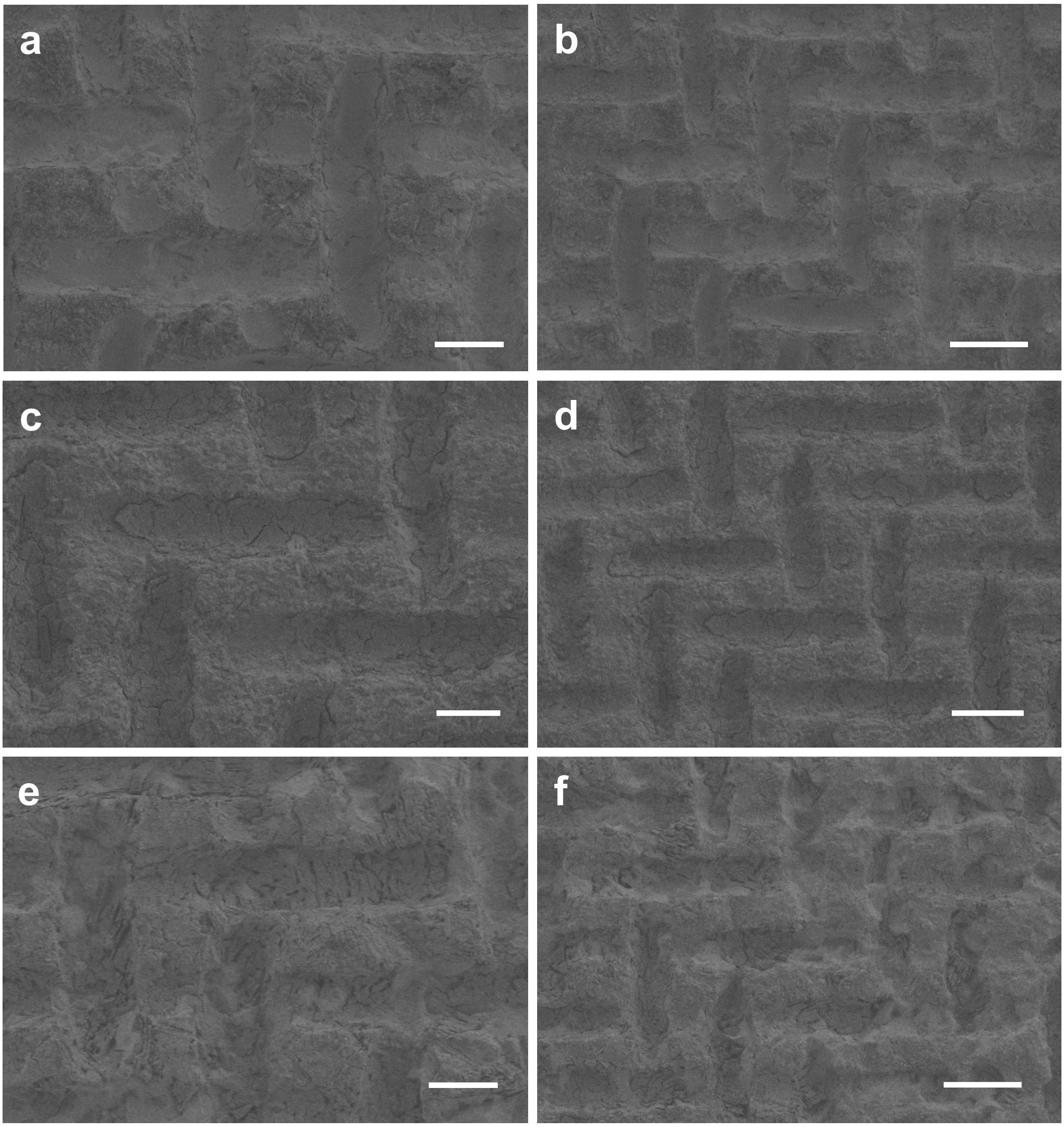

Supplementary Figure 20. SEM images of PVDF-Sn@Zn gradient electrode after **a, b** 100, **c, d** 200 and **e, f** 500 cycles at 5 mA cm^{-2} and 1 mAh cm^{-2}. Scale bar, 30 μm for **a, c, e** and 60 μm for **b, d, f**. The cyclic process starts with plating and ends with stripping.

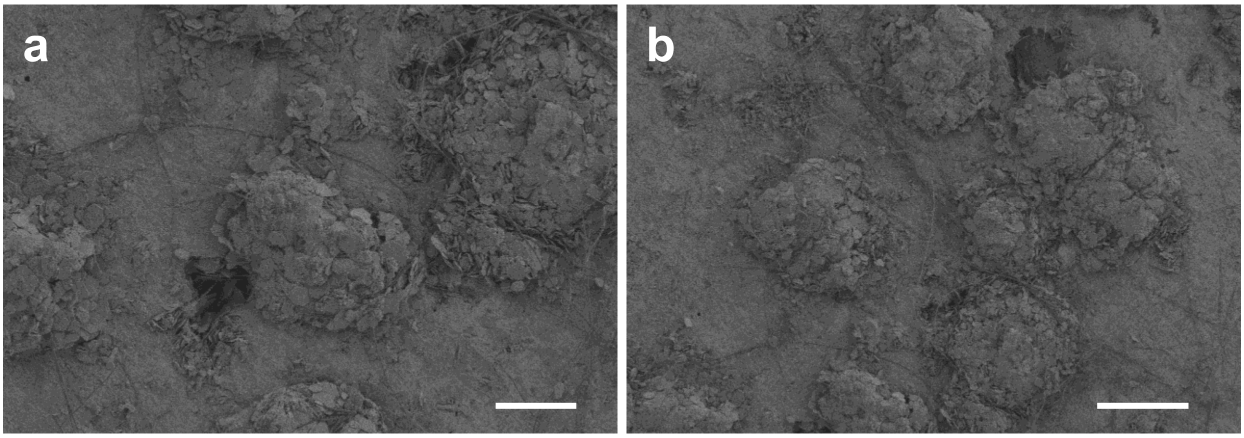

Supplementary Figure 21. SEM images of Zn foil after 100 cycles at 5 mA cm^{-2} and 1 mAh cm^{-2}. Scale bar, 30 μm for **a** and 60 μm for **b**.

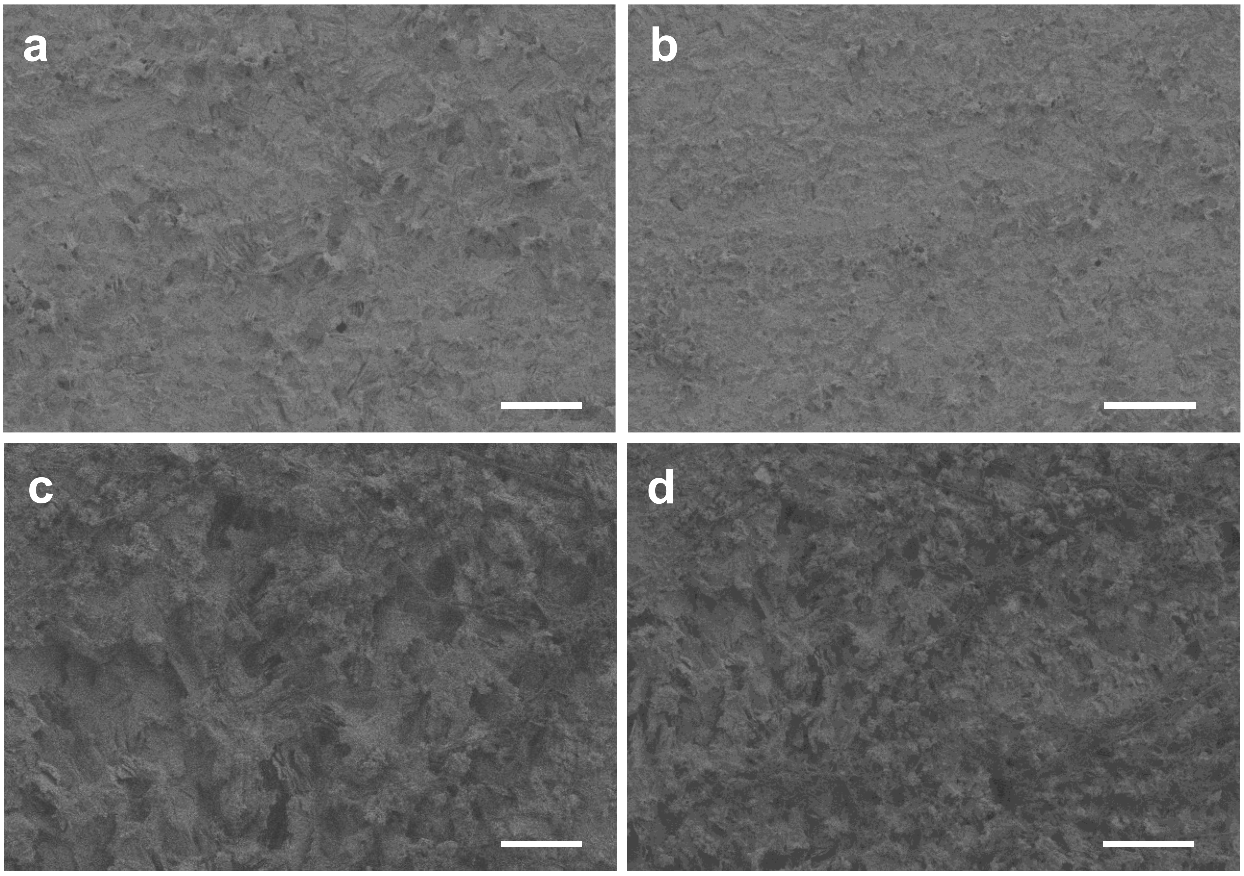

Supplementary Figure 22. SEM images of PVDF@Zn after **a, b** 100 and **c, d** 200 cycles at 5 mA cm^{-2} and 1 mAh cm^{-2}. Scale bar, 30 μm for **a, c** and 60 μm for **b, d**.

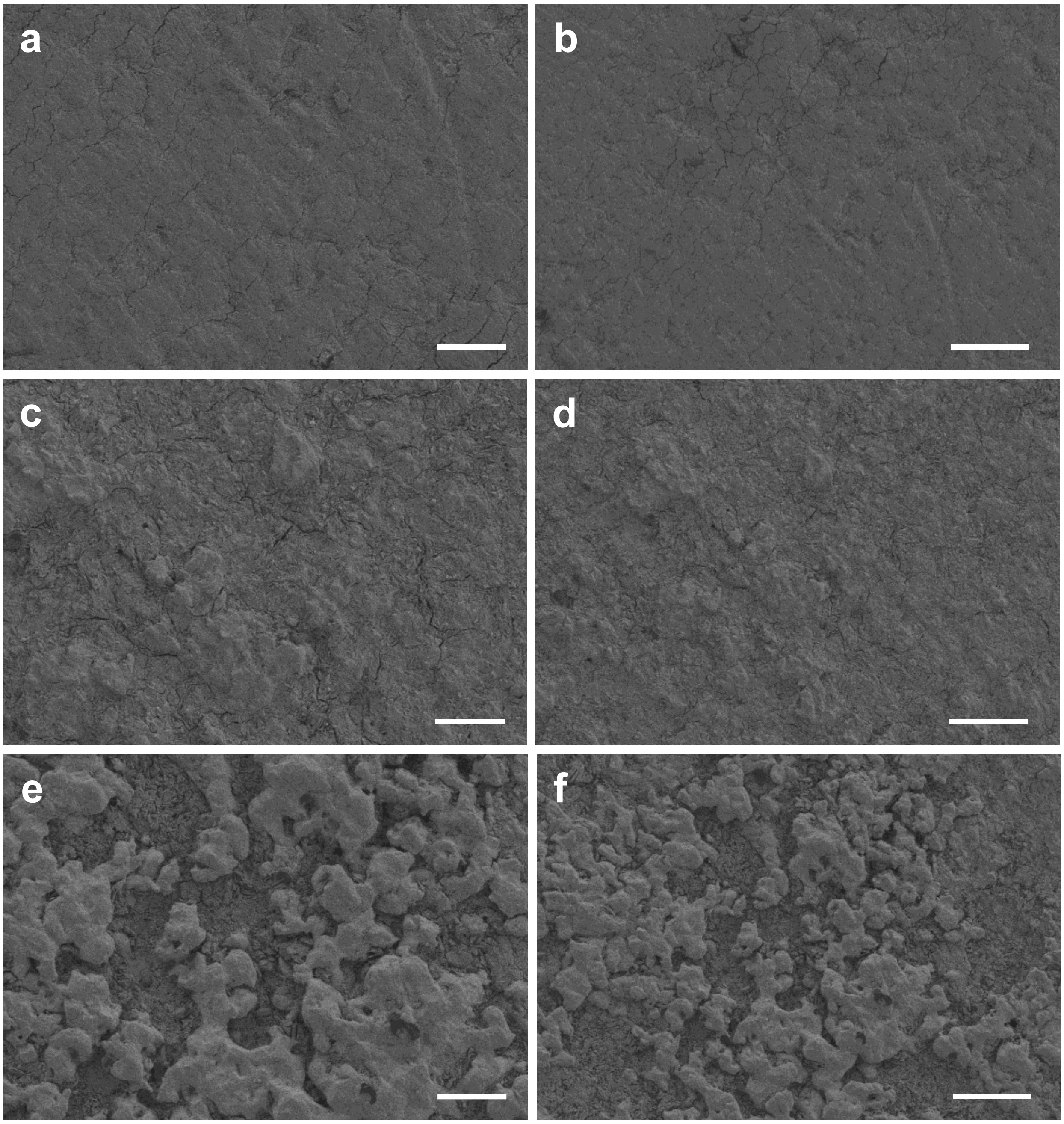

Supplementary Figure 23. SEM images of Sn@Zn electrode after **a, b** 100, **c, d** 200 and **e, f** 400 cycles at 5 mA cm^{-2} and 1 mAh cm^{-2}. Scale bar, 30 μm for **a, c, e** and 60 μm for **b, d, f**.

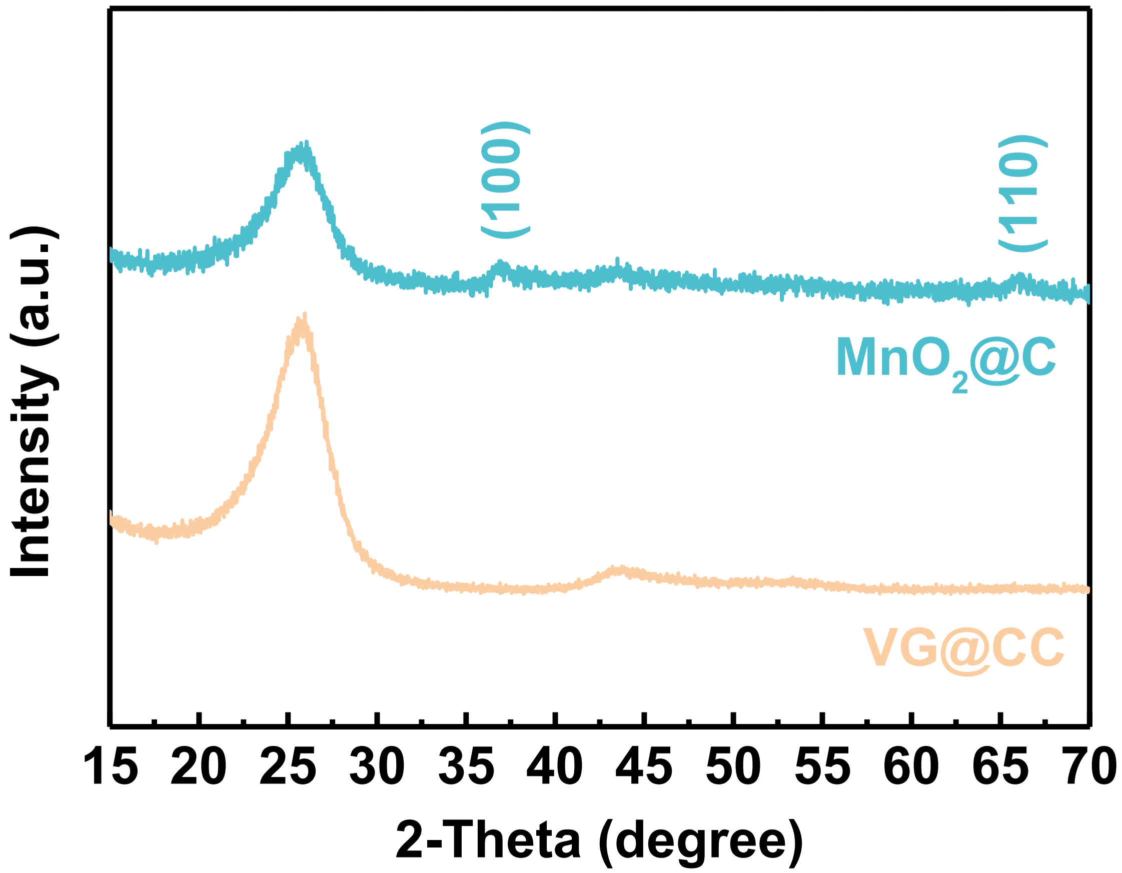

Supplementary Figure 24. XRD of VG@CC and MnO_2@C.

XRD pattern revealed that the obtained products are indexed to pure tetragonal δ-MnO_2 phase (JCPDS 80-1098).

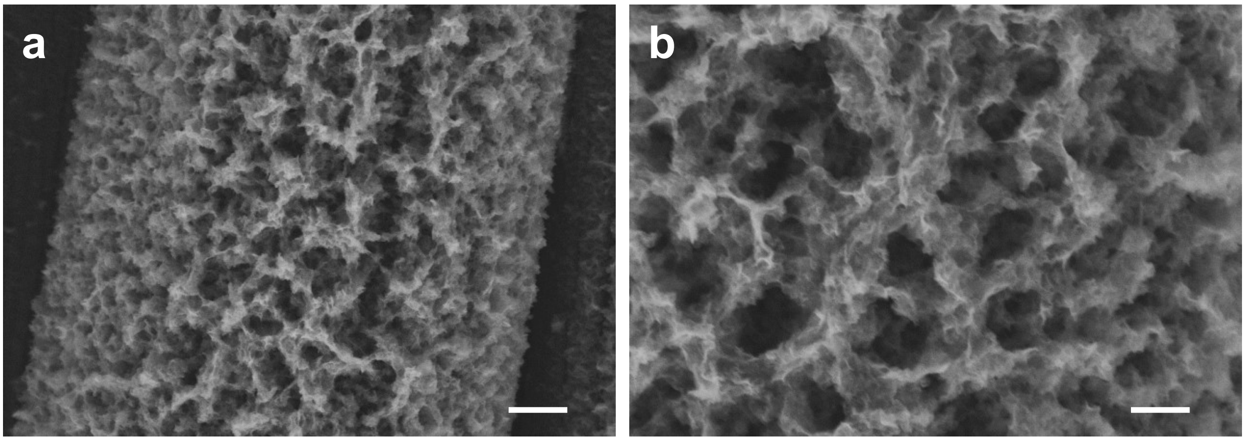

Supplementary Figure 25. SEM images of MnO_2@C. Scale bar, 1 μm for **a** and 500 nm for **b**.

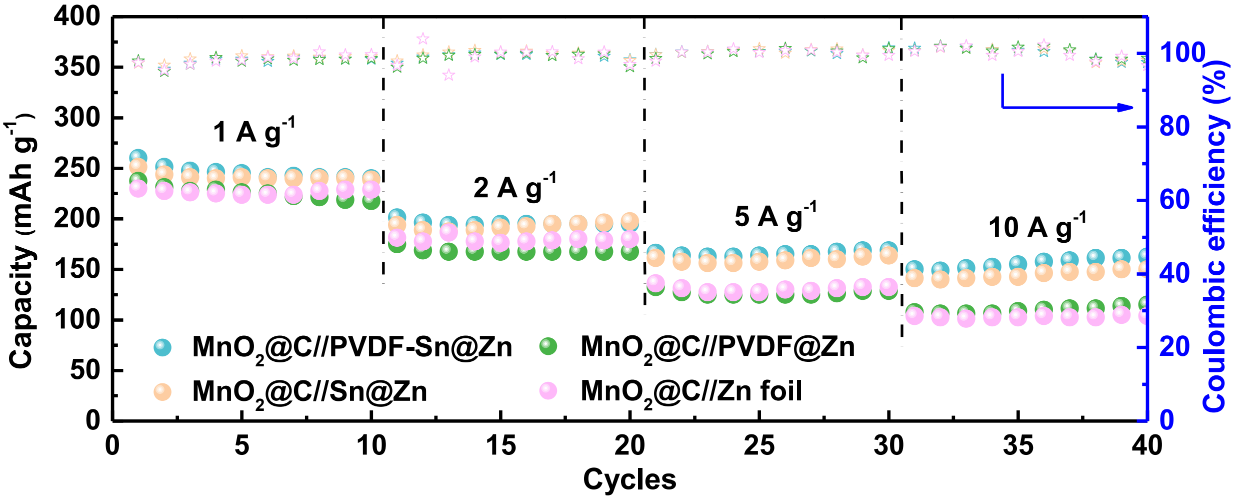

Supplementary Figure 26. Rate performance of different full cells.

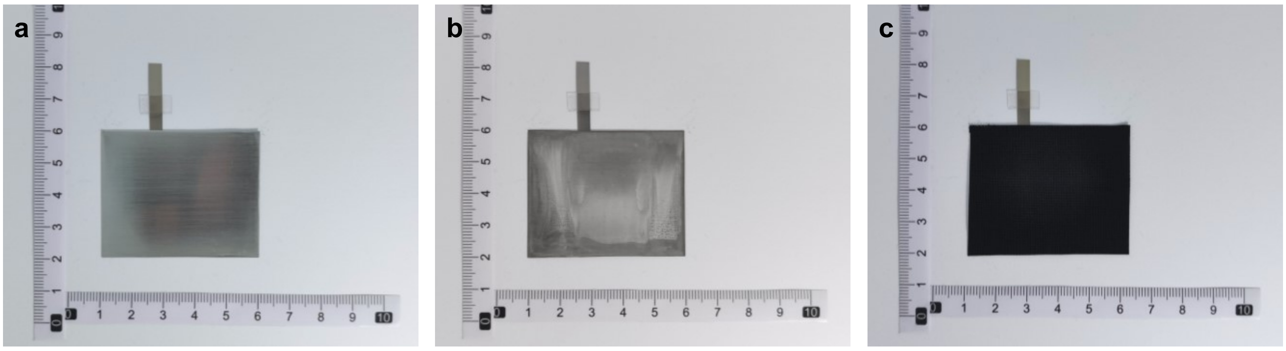

Supplementary Figure 27. Optical photos of **a** Zn foil, **b** PVDF-Sn@Zn and **c** MnO_2@C electrodes with size of 4×5 cm^2.

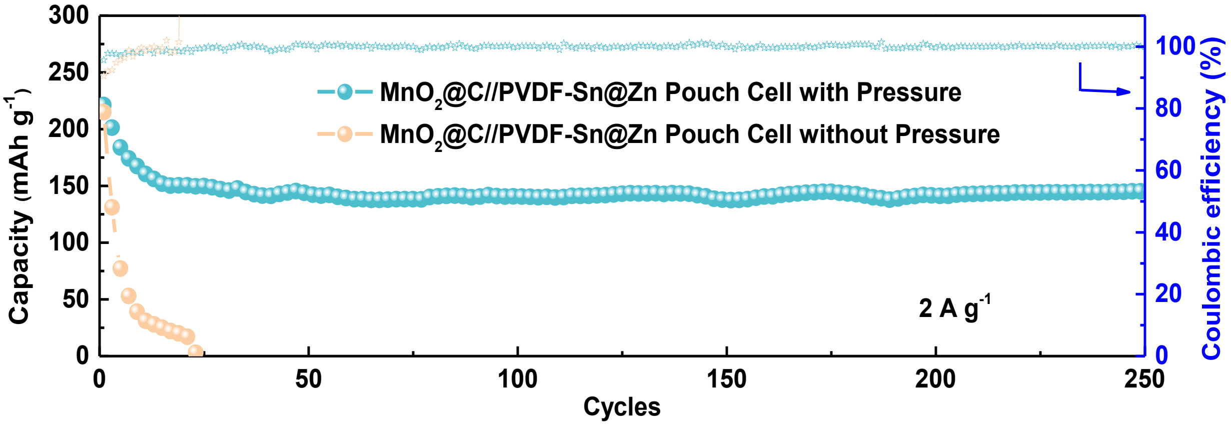

Supplementary Figure 28. The cycling performance of MnO_2@C//PVDF-Sn@Zn pouch cells with/without pressure conditions.

The cycling test of the pouch cells is performed under pressure (by pressing a 50 ml/1.8kg stainless steel reactor against the cell) to ensure better contact between cell components. We have also explored the cycling performance of pouch cells with/without pressure conditions. As shown in Supplementary Figure 28, pouch cell without pressure shows a rapid capacity drop, which can be attributed to poor contact between cell components and high ion/electron transfer resistance. In contrast, pouch cell under pressure exhibits good capacity retention after cycling.

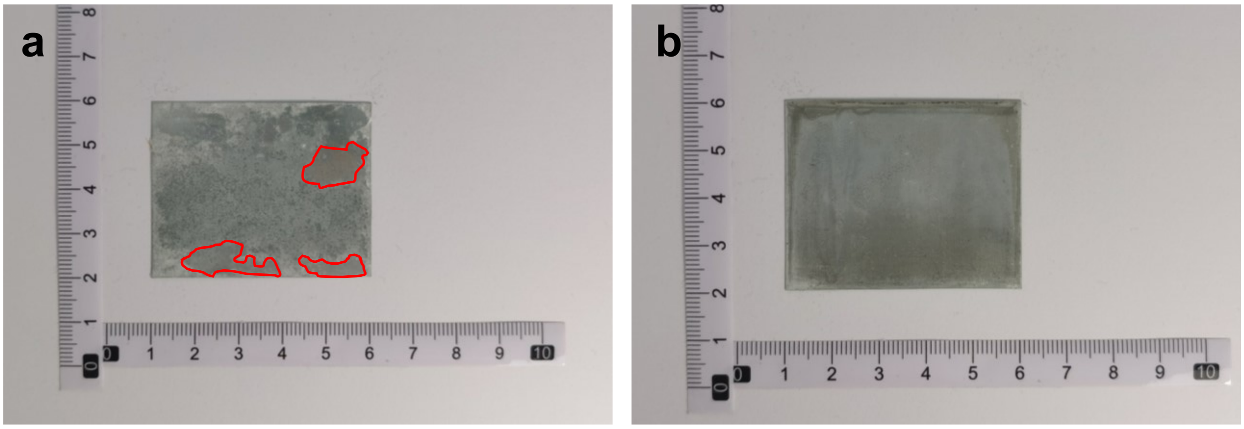

Supplementary Figure 29. Optical photos of **a** Zn foil and **b** PVDF-Sn@Zn after 200 cycles test.

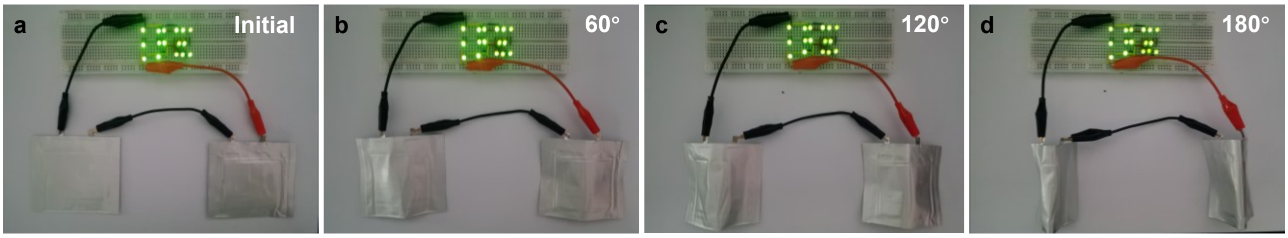

Supplementary Figure 30. Demonstration of flexibility of the MnO_2@C//PVDF-Sn@Zn pouch cell.

Supplementary Table 1. Comparison of voltage hysteresis of reported advanced Zn anodes with this work at the current density/capacity of 1 mA cm^{-2}/1 mAh cm^{-2}.

Zn anode	Voltage hysteresis (mV)	Cycle	Ref.	Published Year
A-Zn	~25	500	ACS nano[1]	2022
Zn@ZnF_2	35.7	800	Adv. Mater.[2]	2021
ZnOHF@Zn	27.2	700	Energy Storage Mater.[3]	2022
Zn@ZnO HPA-2.0	39.3	400	Adv. Funct. Mater.[4]	2020
ZnSn-1	~50	100	Adv. Funct. Mater.[5]	2021
Zn-P-MIEC	> 100	initial	Adv. Mater.[6]	2022
NGO@Zn	17	20	Adv. Mater.[7]	2021
ZF@F-TiO_2	30	100	Nat. Commun.[8]	2020
$Zn_{0.73}Al_{0.27}$@Zn	48.3	initial	Nano Lett.[9]	2022
GFA-5	76	initial	Energy Environ. Sci.[10]	2022
PVDF-Sn@Zn	14.1	500	This work	
	19.4	1000		

Supplementary Table 2. Summary of electrochemical performance of other typical Zn anode based on artificial interfacial layer strategy or 3D structured design.

Zn anode	Current density (mA cm^{-2})	Cycling capacity (mAh cm^{-2})	Cycle life (h)	Ref.	Published Year
Zn@ZnF_2	0.5	1	500	Adv. Mater.[2]	2021
ZnP-MIEC	10	1	100	Adv. Mater.[6]	2022
NGO@Zn	5	5	300	Adv. Mater.[7]	2021
ZF@C-TiO_2	2	2	280	Nat. Commun.[8]	2020
$Zn_{0.73}Al_{0.27}$@Zn	2	2	500	Nano. Lett.[9]	2022
Zn/$Sn_{(200)}$	0.5	1	500	Adv. Mater.[11]	2021
C_{flower}/Zn	5	2.5	150	Nano. Lett.[12]	2022
SDF	3	4.5	250	Adv. Energy Mater.[13]	2021
Sn@NHCF	1	1	370	Sci. Adv.[14]	2022
Zn@ZnO-3D	5	1.25	500	Energy Environ. Sci.[15]	2020
Zn/ex-ZrP	10	10	80	Energy Environ. Sci.[16]	2022
Zn@PFSA	1	1	800	ACS nano[17]	2022
Cu NBs@NCFs-Zn	5	2	250	Adv. Mater.[18]	2022
3D Ni-Zn	5	2	200	Adv. Energy Mater.[19]	2021
PS-Zn	10	1	500	Nat. Commun.[20]	2022
PVDF-Sn@Zn	1<br>5<br>10	1<br>5<br>10	1200<br>500<br>200	This work	

References

1 Yan, Y. et al. Surface-Preferred Crystal Plane Growth Enabled by Underpotential Deposited Monolayer toward Dendrite-Free Zinc Anode. *ACS Nano* **16**, 9150-9162 (2022).

2 Yang, Y. et al. Synergistic Manipulation of Zn^{2+} Ion Flux and Desolvation Effect Enabled by Anodic Growth of a 3D ZnF_2 Matrix for Long-Lifespan and Dendrite-Free Zn Metal Anodes. *Adv. Mater.* **33**, e2007388 (2021).

3 Pan, Z. et al. Zincophilic 3D ZnOHF Nanowire Arrays with Ordered and Continuous Zn^{2+} Ion Modulation Layer Enable Long-term Stable Zn Metal Anodes. *Energy Storage Mater.* **50**, 435-443 (2022).

4 Kim, J. Y., Liu, G., Shim, G. Y., Kim, H. & Lee, J. K. Functionalized Zn@ZnO Hexagonal Pyramid Array for Dendrite-Free and Ultrastable Zinc Metal Anodes. *Adv. Funct. Mater.* **30**, 2004210 (2020).

5 Wang, L. et al. Sn Alloying to Inhibit Hydrogen Evolution of Zn Metal Anode in Rechargeable Aqueous Batteries. *Adv. Funct. Mater.* **32**, 2108533 (2021).

6 Zhang, M. et al. Construction of mixed ionic-electronic conducting scaffolds in Zn powder: A scalable route to dendrite-free and flexible Zn anodes. *Adv. Mater.* **34**, e2200860 (2022).

7 Zhou, J. et al. Ultrathin Surface Coating of Nitrogen-Doped Graphene Enables Stable Zinc Anodes for Aqueous Zinc-Ion Batteries. *Adv. Mater.* **33**, e2101649 (2021).

8 Zhang, Q. et al. Revealing the role of crystal orientation of protective layers for stable zinc anode. *Nat. Commun.* **11**, 3961 (2020).

9 Zheng, J. et al. Electrostatic Shielding Regulation of Magnetron Sputtered Al-Based Alloy Protective Coatings Enables Highly Reversible Zinc Anodes. *Nano Lett.* **22**, 1017-1023 (2022).

10 Liang, G. et al. Gradient fluorinated alloy to enable highly reversible Zn-metal anode chemistry. *Energy Environ. Sci.* **15**, 1086-1096 (2022).

11 Li, S. et al. Toward Planar and Dendrite-Free Zn Electrodepositions by Regulating Sn-Crystal Textured Surface. *Adv. Mater.* **33**, 2008424 (2021).

12 Xu, Z. et al. Efficient Zn Metal Anode Enabled by O,N-Codoped Carbon Microflowers. *Nano Lett.* **22**, 1350-1357 (2022).

13 Shen, Z. et al. Stratified Zinc-Binding Strategy toward Prolonged Cycling and Flexibility of Aqueous Fibrous Zinc Metal Batteries. *Adv. Energy Mater.* **11**, 2100214 (2021).

14 Yu, H. et al. Confining Sn nanoparticles in interconnected N-doped hollow carbon spheres as hierarchical zincophilic fibers for dendrite-free Zn metal anodes. *Sci. Adv.* **8**, eabm5766 (2022).

15 Xie, X. et al. Manipulating the ion-transfer kinetics and interface stability for high-performance zinc metal anodes. *Energy Environ. Sci.* **13**, 503-510 (2020).

16 Peng, H. et al. Intercalation of organics into layered structures enables superior interface compatibility and fast charge diffusion for dendrite-free Zn anodes. *Energy Environ. Sci.* **15**, 1682-1693 (2022).

17 Hong, L. et al. Highly Reversible Zinc Anode Enabled by a Cation-Exchange Coating with Zn-Ion Selective Channels. *ACS Nano* **16**, 6906–6915 (2022).

18 Zeng, Y. et al. Nitrogen-Doped Carbon Fibers Embedded with Zincophilic Cu Nanoboxes for Stable Zn Metal Anodes. *Adv. Mater.* **34**, 2200342 (2022).

19 Zhang, G. et al. 3D-Printed Multi-Channel Metal Lattices Enabling Localized Electric-Field Redistribution for Dendrite-Free Aqueous Zn Ion Batteries. *Adv. Energy Mater.* **11**, 2003927 (2021).

20 Li, Q. et al. Tailoring the metal electrode morphology via electrochemical protocol optimization for long-lasting aqueous zinc batteries. *Nat. Commun.* **13**, 3699 (2022).
